# Development of an Enzyme-Linked Immunosorbent Assay Method Specific for the Detection of G-Group Aflatoxins

**DOI:** 10.3390/toxins8010005

**Published:** 2015-12-28

**Authors:** Peiwu Li, Qian Zhou, Ting Wang, Haiyan Zhou, Wen Zhang, Xiaoxia Ding, Zhaowei Zhang, Perng-Kuang Chang, Qi Zhang

**Affiliations:** 1 Oil Crops Research Institute of the Chinese Academy of Agricultural Sciences, Wuhan 430062, China; peiwuli@oilcrops.cn (P.L.); zhouqianyz513@126.com (Q.Z.); wangting963@163.com (T.W.); zhouhaiyan@caas.cn (H.Z.); zhangwen@oilcrops.cn (W.Z.); dingxiaoxia@caas.cn (X.D.); zhangzhaowei@caas.cn (Z.Z.); 2 Key Laboratory of Biology and Genetic Improvement of Oil Crops, Ministry of Agriculture, Wuhan 430062, China; 3 Key Laboratory of Detection for Mycotoxins, Ministry of Agriculture, Wuhan 430062, China; 4 Laboratory of Risk Assessment for Oilseeds Products (Wuhan), Ministry of Agriculture, Wuhan 430062, China; 5 College of Food Science and Technology, Agricultural University of Hebei, Baoding 071001, China; 6 Southern Regional Research Center, Agricultural Research Service, U.S. Department of Agriculture, New Orleans, LA 70124, USA; perngkuang.chang@ars.usda.gov

**Keywords:** aflatoxin G_1_, class-specific, monoclonal antibody, ELISA, *Aspergillus flavus*

## Abstract

To detect and monitor G-group aflatoxins in agricultural products, we generated class-specific monoclonal antibodies that specifically recognized aflatoxins G_1_ and G_2_. Of the final three positive and stable hybridomas obtained, clone 2G6 produced a monoclonal antibody that had equal sensitivity to aflatoxins G_1_ and G_2_, and did not cross-react with aflatoxins B_1_, B_2_, or M_1_. Its IC_50_ values for aflatoxins G_1_ and G_2_ were 17.18 ng·mL^−1^ and 19.75 ng·mL^−1^, respectively. Using this new monoclonal antibody, we developed a competitive indirect enzyme-linked immunosorbent assay (CI-ELISA); the method had a limit of detection of 0.06 ng·mL^−1^. To validate this CI-ELISA, we spiked uncontaminated peanut samples with various amounts of aflatoxins G_1_ and G_2_ and compared recovery rates with those determined by a standard HPLC method. The recovery rates of the CI-ELISA ranging from 94% to 103% were comparable to those of the HPLC (92% to 102%). We also used both methods to determine the amounts of G-group aflatoxins in five peanut samples contaminated by aflatoxin B_1_-positive, and their relative standard deviations ranged from 8.4% to 17.7% (under 20%), which demonstrates a good correlation between the two methods. We further used this CI-ELISA to assess the ability of 126 fungal strains isolated from peanuts or field soils to produce G-group aflatoxins. Among these, seven stains producing different amounts of G-group aflatoxins were identified. Our results showed that the monoclonal antibody 2 G6-based CI-ELISA was suitable for the detection of G-group aflatoxins present in peanuts and also those produced by fungi.

## 1. Introduction

**Figure 1:**
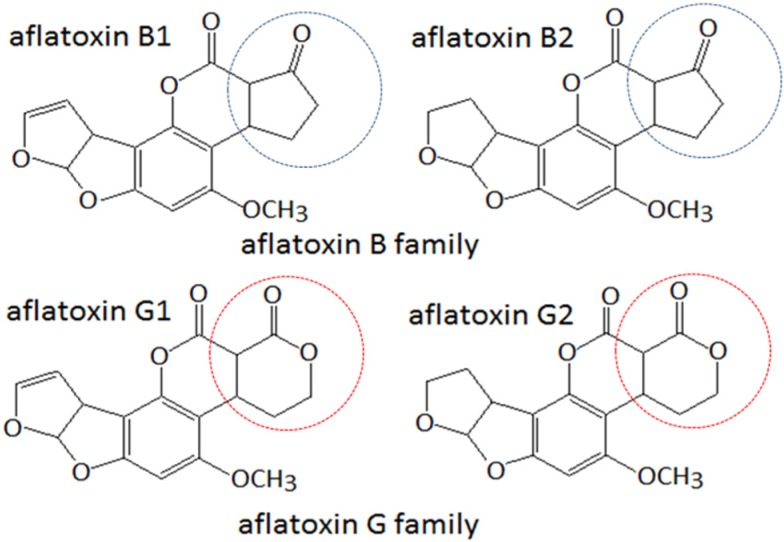
Chemical structures of main aflatoxins divided into two groups, B and G family. The moiety in blue or red Circle shows the difference between the two groups.

Aflatoxins, a group of highly toxic and carcinogenic difuranocoumarin compounds, are secondary metabolites produced by *Aspergillus flavus* and *Aspergillus parasiticus* [1]. Of more than 20 different aflatoxins identified, those belonging to the two major groups, B and G (Figure 1), are frequently found to contaminate food and feed, especially peanuts and maize [2,3,4]. Due to their extremely toxic and carcinogenic effects, legal limits have been imposed on various agri-food products in different countries and regions of the world [5]. For example, the Codex Alimentarius Commission, Joint FAO/WHO Food Standards Program has adopted a limit of 15 μg·kg^−1^ for total aflatoxins [6]. The European Commission sets the maximum level of 0.1–12 μg·kg^−1^ for aflatoxin B_1_ and 4–15 μg·kg^−1^ for total aflatoxins in certain foodstuffs for human consumption [7].

To date, many analytical methodologies have been well-established for the determination of aflatoxins, such as thin layer chromatography (TLC) [8], high-performance liquid chromatography (HPLC) [9], and high performance liquid chromatography-mass spectrometry (HPLC-MS) [10,11]. However, these techniques are time-consuming and not cost-effective, and therefore are not suitable for routine analyses of large numbers of samples. Over the last two decades, the application of immunological methods in aflatoxin detection, particularly enzyme-linked immunosorbent assay (ELISA) and immunochramotographic assay (strip), has been favorably accepted [12,13]. ELISA not only is a tool for rapid and sensitive detection with high sample throughput capacity, but also is relatively inexpensive [13,14]. Production of polyclonal antibodies is simple and easy when enough immunogens and animals are available, but monoclonal antibodies (McAbs) have many other advantages, including the capacity for sustainable production and consistent properties. McAbs against aflatoxins B_1_, M_1_, and G_1_ [15,16,17] have been reported.

Although the toxicity of G-group aflatoxins is lower than that of aflatoxin B_1_, there is still a need to monitor this group of aflatoxins in food and feed to protect health of humans and animals. Of the previously generated three McAbs against aflatoxin G_1_, DE_7_ shows a significant cross-reactivity to aflatoxin B_1_ (8%), 1C_10_ is not sensitive (IC_50_ > 160 ng·mL^−1^), and 1C_8_ exhibits a dominant specificity to aflatoxin G_1_ [17]. In this study, we obtained a novel McAb, which is specific for equal detection of aflatoxins G_1_ and G_2_. With this McAb, a sensitive competitive indirect ELISA was developed.

## 2. Results and Discussion

### 2.1. Immunization, Cell Fusion, and Screening

Immunogen of AFG_1_-BSA significantly induced production of specific antibodies in Balb/c mice. The titers of antibodies of the three mice increased after each immunization, and after the forth immunization the titers reached 1:128,000, 1:64,000, 1:32,000, respectively. The mouse that had the highest titer and cross-reactivity was chosen as the lymphocytes donor for cell fusion. In this study, SP2/0 myeloma cells separated from solid tumor were used for the fusion. We found that the isolated SP2/0 cells increased the fusion rate to approximately 92% with high yields of hybridomas. The results may be mainly due to the high viability of the freshly isolated SP2/0 cells and no microbial (such as fungi) contamination.

Both HAT semi-solid medium and modified two-step ELISA screening methods were used to identify positive monoclonal cells from thousands of hybridomas. The semi-solid medium method facilitated the growth of single slower-growing clones by providing affluent growth factors and cubic space thereby avoiding mix-ups of different hybridomas. After 14 days of cultivation, 201 clones that were screened out by the HAT semi-solid medium were transferred to HT complete medium. Then competitive indirect and non-competitive ELISAs were employed in the two-step screening procedure [15]. Thirty-eight clones whose culture supernatants gave an absorbance value greater than 2.0 were selected by the indirect non-competitive ELISA and transferred to 24-well plates for further screening. Of the 38 clones, three positive clones, 2G6, 3A4, 4G4, that had strong cross-reactivity with aflatoxins G_1_ and G_2_ were picked out after the competitive indirect ELISA screening.

### 2.2. Sensitivity and Specificity

The value of IC_50_ (50% inhibition of binding) and cross-reactivity (CR) are often used to evaluate sensitivity and specificity of a McAb, respectively. The results of sensitivity and specificity of the three clones are summarized in Table 1.

**Table 1:** Sensitivity, specificity, and titer of three monoclonal antibodies against five aflatoxins.

Aflatoxins	2G6			3D4			4A4		
	IC_50_	CR	Titer	IC_50_	CR	Titer	IC_50_	CR	Titer
AFB_1_	- ^a^	0	2.56 × 10^5^	-	0	4 × 10^3^	>500	<5	8 × 10^3^
AFB_2_	-	0		-	0		-	0	
AFG_1_	17.18	100		19.25	100		20.56	100	
AFG_2_	19.75	87		29.62	65		41.96	49	
AFM_1_	-	0		-	0		-	0	

^a^ No inhibition was found in the competitive ELISA. Therefore, no IC_50_ value is available.

The lowest values of IC_50_ of the three McAbs for aflatoxins G_1_ and G_2_ were from clone 2G6, which were 17.18 ng·mL^−1^ and 19.18 ng·mL^−1^, respectively. The CR of 2G6 with aflatoxin G_1_ was 100% similar to that of 3D4 and 4A4, and its CR with aflatoxin G_2_ was 87%, the highest among the three clones. In contrast, the CR of all three with AFB_1_, AFB_2_, and AFM_1_ was well below 5%. This probably results from different electronegativity and geometry of the heterocyclic structure (see Figure 1), which was described previously [18]. 2G6 distinguished G-group aflatoxins from B-group aflatoxins; it had a sensitivity comparable to that reported previously (IC_50_ for AFG_1_ was 13.92 ng·mL^−1^) [17] but a much higher CR. We determined 2G6 to be in the same subclass of IgG_2a_. Up to now, it is the best class-specific (recognizing a group of compounds rather than one) McAb against G-group aflatoxins. Therefore, we selected 2G6, which also is the most stable clone, for further optimization of the CI-ELISA.

### 2.3. Optimization of CI-ELISA

**Figure 2:**
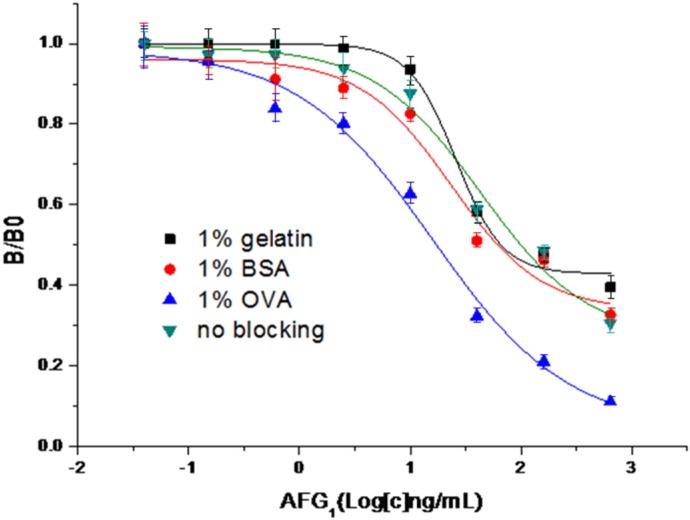
Effects of different blocking solvents on sensitivity of CI-ELISA. IC_50_ values for aflatoxin G_1_ are as follows: 65.61 ng·mL^−1^ (1% gelatin), 61.24 ng·mL^−1^ (1% BSA), 17.18 ng·mL^−1^ (1% OVA), and 98.98 ng·mL^−1^ (no blocking). Maximum optical densities were about 1.0.

The ELISA optimization was performed using aflatoxin G_1_ as the competitor analyte. Based on the preliminary experimental parameters of coating antigen (2 μg·mL^−1^) and antibody dilution (1:32,000), we further optimized other parameters of blocking buffer, organic solvent concentration, and reaction buffer to increase sensitivity of the CI-ELISA. Figure 2 shows that 1% ovalbumin (OVA) gave the highest sensitivity of 17.18 ng·mL^−1^ compared to others (65.61 ng·mL^−1^ for 1% gelatin, 61.24 ng·mL^−1^ for 0.5% BSA and 98.98 ng·mL^−1^ for no blocking). The result suggests that 1% OVA has the weakest impact on the reaction of hapten-antibody.

**Figure 3:**
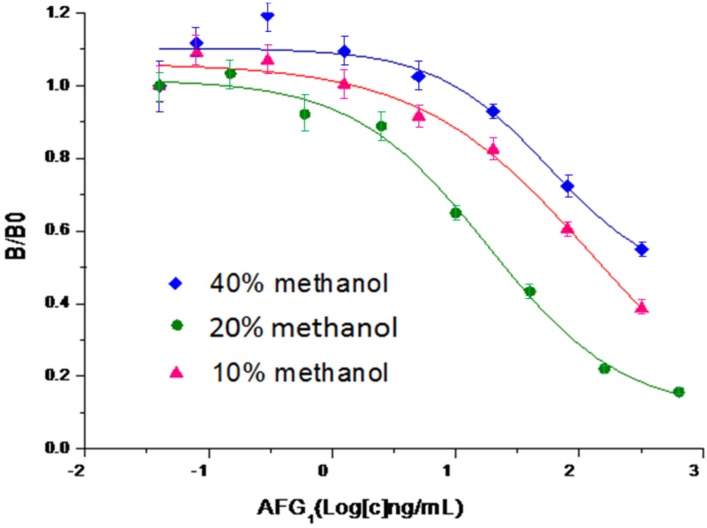
The effect of methanol concentration on sensitivity of CI-ELISA. IC_50_ values for aflatoxin G_1_ are as follows: 195.6 ng·mL^−1^ (40% methanol), 24.98 ng·mL^−1^ (20% methanol) and 155.7 ng·mL^−1^ (10% methanol). Maximum optical densities were about 1.0.

Figure 3 shows that the use of 20% methanol gave the highest sensitivity with a IC_50_ value of 24.98 ng·mL^−1^. In general, a methanol concentration higher than 10% decreases the activity of antibodies thus decreasing the assay sensitivity. However, in our case, the improved sensitivity may result from the better dissolving ability of 20% methanol of aflatoxin G_1_. Finally, the reaction buffer of PBST, which gave the lowest IC_50_ value of 17.18 ng·mL^−1^, was selected (data not shown).

Using these optimized conditions, similar curves were observed for aflatoxin G_2_. Thus, we established a standard curve of CI-ELISA for G-group aflatoxins. The average value of IC50 was 17.18 ng·mL^−1^ and the limit of detection was 0.6 ± 0.02 ng·mL^−1^.

### 2.4. Analysis of Spiked Samples and Naturally Contaminated Samples

For the spike-recovery study, peanut samples that were free of aflatoxins were deliberately contaminated with 1 mL of G-group aflatoxins (G_1_ + G_2_) working solutions of concentrations of 5, 50, and 200 ng·mL^−1^ (Table 2). Since matrix interference is a common problem for all aflatoxin-specific immunoassays, dilution of the sample extracts was performed to reduce the interference [19].

**Table 2:** Recovery rates of G-group aflatoxins in peanuts with CI-ELISA and HPLC.

Theoretical (ng·mL^−1^) ^a^	CI-ELISA			HPLC		
	Measured (ng·mL^−1^)	Recovery (%)	Mean ± SD (%)	Measured (ng·mL^−1^)	Recovery (%)	Mean ± SD (%)
1	1.05	105.0	101 ± 4.6	0.96	96.2	92.2 ± 3.0
	0.96	96.0		0.89	89.1	
	1.02	102.0		0.91	91.3	
10	9.59	95.9	94.2 ± 3.3	9.64	96.4	96.3 ± 1.6
	8.96	89.6		9.43	94.3	
	9.70	97.0		9.81	98.1	
40	41.32	103.3	102.8 ± 2.6	41.79	104.5	101.7 ± 3.1
	42.28	105.7		39.01	97.5	
	39.79	99.5		40.98	102.5	

^a^ Peanuts were spiked with indicated amounts of aflatoxins G_1_ and G_2_ (weight ratio is 1:1). The values correspond to the spiked amounts in the working solutions after being diluted with five volumes of PBS (see Experimental Section 3.9 for details).

The recovery rates of the developed CI-ELISA ranged from 94% to 103%, which conform to the recommended recovery rate (70%–110%) of Commission Regulation about mycotoxins in foodstuff [7]. The spiked amounts of G-group aflatoxins in the peanut samples were also determined by a standard HPLC method, which gave recovery rates ranging from 92% to 102%. These results demonstrate that the developed CI-ELISA is suitable for the detection of G-group aflatoxins in peanuts. To further confirm the utility of this CI-ELISA method, we used it to detect and determine the amounts of G-group aflatoxins in five contaminated (aflatoxin B_1_-positive) peanut samples collected in the last three years. We compared the results with those obtained from the aforementioned HPLC method. Table 3 shows the relative standard deviations (RSD) ranging from 8.4% to 17.7% (under 20%), which indicates a good correlation between the two methods.

**Table 3:** Detection of G-group aflatoxins in the peanut samples with CI-ELISA and HPLC.

Samples	CI-ELISA (μg·kg^−1^)	HPLC (μg·kg^−1^)			Two Methods RSD (%)
	AFG Group Mean ± SD	AFG_1_ Mean ± SD	AFG_2_ Mean ± SD	AFG_1_ + AFG_2_	
1	12.6 ± 1.3	9.8 ± 0.7	No	9.8	17.7
2	17.3 ± 1.5	13.5 ± 1.0	1.2 ± 0.1	14.7	11.5
3	16.0 ± 1.3	13.3 ± 0.6	0.9 ± 0.1	14.2	8.4
4	n.d. ^a^	n.d.	n.d.	n.d.	-
5	n.d.	n.d.	n.d.	n.d.	-

^a^ n.d. = not detected, limits of detection of ELISA and HPLC for were 0.75 and 1 μg·kg^−1^ respectively.

### 2.5. Identification of Aspergillus Strains that Produce G-Group Aflatoxins

**Figure 4:**
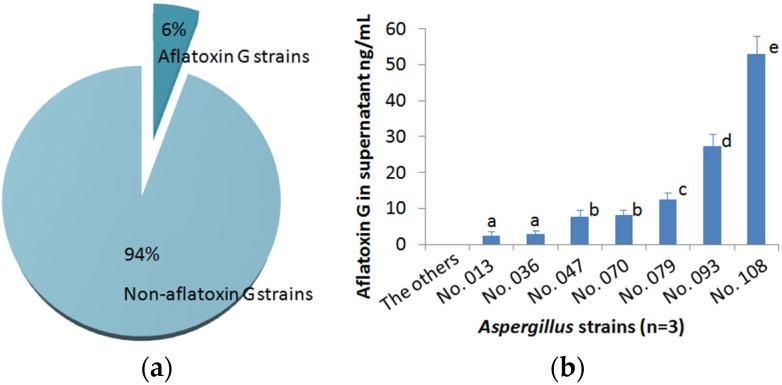
Identification of *Aspergillus* strains producing G-grous aflatoxins. (**a**) Proportions of G-group-aflatoxin-producing and non-producing *Aspergillus* isolates; (**b**) Amounts of G-group aflatoxins in the supermatnats of PDB cultures determined by CI-ELISA, showing five significantly different levels with a statistical analysis (*p* < 0.05).

*A. flavus* and *A. parasiticus* are morphologically similar and both produce B-group aflatoxins. In addition, *A. parasiticus* produces G-group aflatoxins [1]. These two species are frequently isolated from agricultural commodities and fields. We examined a total of 126 putative *A. flavus* strains previously isolated from peanuts or field soil samples by the CI-ELISA method to determine if some isolates are *A. parasiticus*. To this end, we assayed supernatants of respective PDB cultures for G-group aflatoxins. The result showed that seven isolates (6%) produced various amount of G-group aflatoxins (Figure 4), which suggests that these isolates likely are *A. parasiticus*. However the identification of a fungal species through the chemical analysis of mycotoxins is quite hazardous. For further strain identification, molecular analysis (PCR) should be performed.

## 3. Experimental Section

### 3.1. Chemicals and Instruments

Aflatoxin G_1_ (solid), aflatoxin G_2_, B_1_, B_2_, and M_1_ standard solutions, Freund′s complete adjuvant and Freund’s incomplete adjuvant, bovine serum albumin (BSA), ovalbumin (OVA), goat anti-mouse immunoglobulin G horseradish peroxidase (IgG/HRP), 3,3,5,5-*tetra*-methylbenzidine (TMB), hypoxanthine aminopterin thymidine (HAT), hypoxanthine thymidine (HT), polyethylene glycol 1450 (PEG 1450, 50%), and mouse monoclonal antibody ISO2-1 kit were purchased from Sigma-Aldrich (St. Louis, MO, USA). Culture media RPMI-1640 with l-glutamine and HEPES (free acid, 238.3 g·L^−1^) was obtained from Thermo Hyclone (Logan, UT, USA). Penicillin (+10,000 Units per milliliter) and streptomucin (+10,000 μg·mL^−1^) was from Gibco (Grand Island, NY, USA). Hybridoma Fusion and Cloning Supplement (HFCS) was obtained from Invitrogen (Waltham, MA, USA). All chemicals and organic solvents were analytical reagent grade. Female Balb/c mice of 8–10 weeks old were purchased from Wuhan Institute of Biologic Products (Wuhan, China). SP2/0 myeloma cells were purchased from China Center for Type Culture Collection (CCTCC, Wuhan, China). Cell culture plates (6, 24, and 96 wells) were from Shanghai Sunub Bio-Tech Development, Inc. (Shanghai, China) Cell culture flasks were from Iwaki Co. (Iwaki, Japan). 96-well polystyrene microplates were from Costar (Cambridge, MA, USA). A microtiter plate reader of Spectra Max M2e with a computer-controlled system was from PerkinElmer (Waltham, MA, USA). HPLC series (Agilent 1100, Agilent Technologies, Santa Clara, CA, USA) consisted of a fluorescence detector, a C18-column (5 µm particle size, 150 mm × 4.6 mm I.D., Agilent Technologies, Santa Clara, CA, USA), and a post-column derivation system.

### 3.2. Solutions, Buffers, and Growth Media

The following solutions and media were used: (1) stock solutions of aflatoxin B_1_, B_2_, G_1_, G_2_, and M_1_ at a concentration of 1 μg·mL^−1^ were prepared in methanol and diluted to standard solution with PBS; (2) coating buffer was 0.1 M carbonate buffer, pH 9.6; (3) phosphate buffer saline (PBS), pH 7.4; (3) washing buffer was 0.05% Tween20 (*v*/*v*) in PBS (PBST); (4) blocking buffer was 1% OVA (*w*/*v*) in PBST; (5) substrate solution was freshly prepared with 10 mL pH 5.0 phosphate-citrate buffer, 1 mL 2 mg·mL^−1^ TMB (in ethanol) and 25 μL 3% H_2_O_2_; (6) stop solution was 2 mol·L^−1^ H_2_SO_4_; (7) complete medium was 78 mL RPMI-1640 medium, 20 mL fetal bovine (inactivated at 56 °C for 30 min), 1 mL antibiotics (+10,000 Units per milliliter penicillin, +10,000 μg per milliliter streptomucin) and 1 mL HEPES; (8) freezing solution was 10% (*v*/*v*) dimethyl sulphoxide (DMSO) in complete medium.

### 3.3. Preparation of Artificial Antigen

Aflatoxin G_1_ was conjugated with BSA and OVA to yield immunogens and coating antigens, respectively, according to the method of reductive alkylation [20]. Briefly, 2.5 mg of aflatoxin G_1_ was dissolved in 2.1 mL acetone, then 30 μL of 10% H_2_SO_4_ was added into the solution. After the mixture was stirred with a magnetic bar at 60 °C for 5 h, 1.5 mL of Milli-Q water was added. The acetone in the mixture was removed with a vacuum rotary evaporator, and the product was extracted with chloroform (5 mL × 4) and washed with 4.5 mL of Milli-Q water. After the removal of the chloroform, the resulting powder was deep yellow. The powder (0.6 mg) was added into a PBS buffer containing 10 mg BSA (or OVA) and reacted at 37 °C for 30 min. After that, 70 μL of 1 mg/mL NaBH_4_ in water was added. The reaction was continued for another 30 min at 4 °C and then stopped by adding 35 μL of 0.1 N HCl. The conjugates were dialyzed against two liters of PBS for three days (two changes of buffer each day). The AFG_1_-BSA conjugate was stored at −20 °C and the AFG_1_-OVA conjugate was mixed with an equal volume of glycerol and stored at −20 °C until use.

### 3.4. Immunization

Three female Balb/c mice were subcutaneously immunized with AFG_1_-BSA in multiple-site. In the first immunization, 100 μg of immunogen was dissolved in 200 μL sterilized PBS and then emulsified with an equal volume of complete Freund’s adjuvant to obtain the final water-in-oil emulsion. Three subsequent subcutaneous injections were followed on days 22, 43, and 64 with emulsion of the equal volume of Freund’s incomplete adjuvant. Blood was drawn from the tail of each mouse after eight days of each immunization and centrifuged to obtain the sera. The sera were assayed for the antibody titer against AFG_1_ by indirect ELISA and for properties of analyte recognition by competitive indirect ELISA. Three days before cell fusion, the mouse with the highest antibody titer was given an intraperitoneal booster injection of 200 μg of AFG_1_-BSA without any adjuvant and sacrificed to be spleen donor for hybridoma production.

### 3.5. Cell Fusion and Cloning

SP2/0 myeloma cells were injected subcutaneously into a free Balb/c mouse to produce SP2/0 solid tumor. The SP2/0 myeloma cells were collected aseptically from the solid tumor and homogenized in a glass homogenizer. The cell supernatant was separated by density gradient centrifugation with lymphocyte separation medium, and then stored at 4 °C in RPMI 1640 medium for later use. Cell fusion was carried out according to the conventional method [21]. The mouse of interest was sacrificed and spleen cells were aseptically harvested. Spleen cells (10^8^) were fused with SP2/0 myeloma cells (10^7^) at a ratio of 5–10:1 in the presence of PEG1450. The fused cells were suspended in 20 mL of complete medium and then mixed with 80 mL semi-solid complete medium containing 1% HAT, methylcelluose, hybridoma fusion and cloning supplement (HFCS), and non-essential amino acids. The fused cells were evenly distributed into six-well plates and incubated in 5% CO_2_ at 37 °C. After 12 days of cultivation, many tips of monoclonal hybridomas that could be seen by naked eyes in the semi-solid medium were single picked out to 96-well culture plates containing 1% HT complete medium. Culture supernatants from each well were screened when the hybridomas reached half-confluence (15–17 days) [22]. The hybridomas whose culture supernatants gave an absorption value of over 2.0 by indirect noncompetitive ELISA were transferred to 24-well microculture plates. Supernatants from 24-well plates were tested again both in indirect noncompetitive and competitive ELISA. Only those hybridomas that retained high absorption values and had good cross-reactivity with aflatoxins G_1_ and G_2_ were selected for further screening. Hybridomas of interest were cultivated for three months for detecting the stability then propagated, and stored in freezing solution according to the freezing procedure: 30 min at 4 °C, overnight in gas of liquid nitrogen and cryopreserved in liquid nitrogen.

### 3.6. ELISA Screening

The indirect noncompetitive ELISA procedure [15] was carried out with minor modifications. AFG_1_-OVA of 2 μg·mL^−1^ was diluted with coating buffer, coated onto microtiter plates (100 μL per well), and incubated overnight at 4 °C. The coated plates were washed three times with PBST and blocked with blocking buffer for 2 h at 37 °C (200 μL per well). After an additional wash, 100 μL of supernatant was added to each well and incubated for 2 h at 37 °C. Following the same washing step, 100 μL per well of IgG-HRP solution (1:20,000 in PBST) was added and incubated for 1 h at 37 °C. After the sixth wash, color development was carried out by adding freshly prepared substrate solution (100 μL per well), gentle shaking, and incubation for 15 min at 37 °C [23]. Stop solution (50 μL) was added to each well to stop the enzymatic reaction. The absorbance was measured at 450 nm with a microplate reader. The competitive indirect ELISA procedure was carried out to screen clones that had specificity for G-group aflatoxins. This method was similar to that of indirect ELISA, except that mixing the same volume of aflatoxin standard solutions and of supernatant (50 μL: 50 μL) was done before adding the mixture to each well [24].

### 3.7. Determination of Isotype, Sensitivity, and Specificity

Ascetic fluids were prepared according to the method described previously [25] with minor modifications. The fluids were purified by octanoic acid-octanoic acidoctanoic acidoctanoic acidammonium sulfate precipitation and dialyzed with deionized water for three days. The isotype of each monoclonal antibody was determined by a commercially available isotyping kit from Sigma (St. Louis, MO, USA).

The competitive indirect ELISA format described above was carried out to evaluate the sensitivity of monoclonal antibodies. Standard solutions of aflatoxin B_1_, B_2_, G_1_, G_2,_ M_1_, with eight concentrations ranging from 0.125 to 640 ng·mL^−1^ were prepared. The absorbance at each aflatoxin concentration and the absorbance without aflatoxin were referred as B and B0, respectively. Sigmoidal curves were plotted as inhibitions (B/B0) versus logarithm of analyte concentration. The cross-reactivities (CRs) of antibody for different analogies were measured with the IC_50_ values (analyte concentration resulting in half-maximum inhibition) and calculated as follow: CR (%) = (IC_50_ AFG_1_/IC_50_ analyte) × 100.

The indirect noncompetitive ELISA was carried to determine the titer. The ELISA titer of ascetic fluid was defined as the highest ascetic dilution which gave an absorbance greater than the pre-immuned control serum in the first dilution.

### 3.8. Optimization of CI-ELISA

Initial experiments were performed to assess the optimal working concentrations of coating antigen and antibody. Different concentrations of coating antigen (0.5, 1, 2, and 4 μg·mL^−1^) and antibody dilutions (1:16,000, 1:32,000, 1:64,000, 1:128,000) were used in combination and optimized by comparing the resulting dose-response curves. According to an optimized experimental design [26], the optimal combination was selected when optical density was about 1.0.

With the optimized working concentrations of coating antigen and antibody, a series of parameters including blocking buffer, organic solvent concentration, and reaction buffer were evaluated to improve the sensitive of CI-ELISA [27]. The optimum conditions were determined by IC_50_, which is the most significant criterion for the immunoassay. The effect of organic solvent (10%, 20%, and 40% of methanol), blocking buffer (1% OVA, 1% BSA, 1% gelatin and without blocking agent) and reaction buffer (PBS and PBST) on sensitivity of ELISA was studied sequentially. Based on the optimal conditions, CI-ELISA using different aflatoxin G_1_ standards (0.125–640 ng·mL^−1^) was repeated five times to establish quantitive standard curve for aflatoxin G_1_ and evaluate the precision and sensitivity.

### 3.9. Analysis of Peanut Samples Spiked with and Naturally Contaminated by Aflatoxin G_1_

The spike and recovery study was carried out by spiking uncontaminated peanuts samples with different amounts of G-group aflatoxins (G_1_ + G_2_) [19,28]. Three working concentrations (5, 50, and 200 ng·mL^−1^) were prepared. Each peanut sample (cut into pieces) 1.0 g in 20-mL glass tube was added with 15, 150, or 600 ng the analytes (spiking levels were 15, 150, and 600 ng·g^−1^ respectively). And then the spiked samples were extracted with an 80% methanol (*v*/*v*) solution that contained 4% (*w*/*v*) NaCl and treated by ultrasound for 5 min. The mixture was allowed to stand for 30 min to separate the supernatant. Each clear supernatant was diluted in five volumes of PBS and analyzed by both the developed CI-ELISA and a HPLC equipped with a fluorescence detector (wavelength of excitation 360 nm and emission 420 nm), which is the Chinese detection standard method [29].

### 3.10. Identification of G-Group Aflatoxin-Producing Fungal Strains

The 126 *A. flavus/A. parasiticus* isolates collected from peanuts or field soil samples were provided by Oil Crops Research Institute of the Chinese Academy of Agricultural Sciences. Spore suspensions were prepared by flooding 10-day-old PDA cultures with sterile distilled water containing 0.1% Tween 80. Spores were counted with a haemocytometer and were diluted with sterile distilled water as required. Fungal cultures were grown in the PDB medium for 14 days. Each culture broth was diluted with four volumes of PBS buffer (pH 7.4) and assayed by the developed CI-ELISA method.

## 4. Conclusions

We successfully obtained three monoclonal antibodies 2G6, 3A4, 4G4 against G-group aflatoxins. The equally high sensitivity of 2G6 toward aflatoxin G_1_ and aflatoxin G_2_ renders it the best class-specific monoclonal antibody candidate for the development of ELISA. The validity and utility of the developed method are evidenced by the recovery rates of spiked and naturally occurring G-group aflatoxins in peanuts. These recovery rates are comparable to those determined by a standard HPLC method, and the two methods show a high degree of correlation. Therefore, the developed ELISA provides an alternative for detecting and monitoring G-group aflatoxins as well as their producing fungal strains that contaminate agricultural commodities to ensure the safe supply of food and feed.
